# Analysis of the coupling coordination between traditional Chinese medicine medical services and economy and its influencing factors in China

**DOI:** 10.3389/fpubh.2024.1320262

**Published:** 2024-06-04

**Authors:** Huan Liu, Zicheng Jiang, Jing Deng, Dexun Li

**Affiliations:** ^1^School of Hospital Economics and Management, Anhui University of Chinese Medicine, Hefei, China; ^2^Key Laboratory of Data Science and Innovative Development of Chinese Medicine in Anhui Province Philosophy and Social, Hefei, China

**Keywords:** traditional Chinese medicine medical services, economic, coupling coordination, Tobit, influence factor

## Abstract

**Background:**

The coordinative relationship between medical treatment and the economy is an important part of promoting the sustainable and healthy development of a social economy, and it is conducive to promoting the coordinated development of the two systems.

**Methods:**

Based on constructing a comprehensive evaluation index system for the relationship between traditional Chinese medicine medical services and economic development, the entropy method, coupling coordination model, and Tobit model are used to calculate the comprehensive development level, coupling coordination degree, and influencing factors of the two systems in 31 provinces of China from 2015 to 2021. Suggestions are proposed for the coordinated development of traditional Chinese medicine medical services, and economy.

**Results:**

The results showed that the development level of traditional Chinese medicine was higher than that of social and economic development, the relative development gap between the two systems was gradually narrowed, and the coupling coordination degree increased steadily; however, the regional differences were large. The relative development level and coupling coordination degree of each region show a development pattern of eastern > central > western > northeast; the coupling coordination degree of the two systems in the spatial region was positively correlated and was enhanced year by year. From the perspective of influencing factors, the level of economic development is the decisive factor in the coupling and coordination between traditional Chinese medicine medical treatment and the social economy. Factors such as human capital and health level in traditional Chinese medicine have an inhibitory effect on the coordinated development of the two systems.

**Conclusion:**

So, China should focus on optimizing the regional allocation of traditional Chinese medicine medical personnel, improving the quality of medical services, and enhancing the health level of medical personnel.

## 1 Introduction

Chinese traditional medicine has five major resource advantages in health, economy, technology, culture, and ecology (1), making indelible contributions to the lives and health of working people in China and around the world. In 2016, the State Council of China proposed in the “Outline of the Strategic Plan for the Development of Traditional Chinese Medicine (2016–2030)” that by 2030, all traditional Chinese medicine services will be covered, and greater contributions will be made to economic and social development (2). It can be seen that the development of traditional Chinese medicine services concerns people's livelihood, and the Chinese government has been emphasizing the role of traditional Chinese medicine development in the social economy. China is facing a critical period of comprehensive construction of socialist modernization, adhering to the development concepts of innovation, coordination, green, openness, and sharing. However, the problem of imbalanced and insufficient development of traditional Chinese medicine services in China is still prominent, with problems such as unreasonable allocation of traditional Chinese medicine resources. At present, sustainable development has become an issue of the utmost importance across the world, especially after the proposed “Healthy China strategy” (3); the coordinated relationship between traditional Chinese medicine medical treatment and the social economy deserves great attention.

According to the literature review, the study on the interaction between medical and health services and the economy in some developed countries is relatively preliminary, mainly exploring the relationship between medical and health expenditure and gross domestic product (GDP) growth. Since Joseph Newhouse noticed a strong positive correlation between per capita health expenditure and per capita GDP, many scholars have devoted themselves to studying the positive correlation or causal relationship between health expenditure and economic development. Some studies have shown that improvements in health can lead to an increase in GDP and vice versa (4, 5). Hitiris and Posnett (6) used a large number of time series and cross-sectional observations to re-examine the results of previous studies, which confirmed the importance of GDP as a determinant of health expenditure. Mayer (7) studied whether there is a Granger causality between medical expenditure and income in 18 Latin American countries and found that there is a significant causal relationship between income and medical expenditure. Raghupathi (8) explored the relationship between public health spending and economic performance across the United States and found that citizens' health status does lead to an overall improvement in economic conditions. Wang (9) proposed that in an economically developed country, there is a high demand for medical services, and the level of medical services should meet the medical needs. Subsequently, some scholars have studied the relationship between medical and health expenditure and economic development in developing countries, and the results generally reflect that medical and health expenditure is crucial to developing countries (10–12), and there is an iterative relationship between economic growth and health-economic growth leading to human capital investment and health progress. Good population health leads to higher labor productivity and economic growth (8, 13). In developing countries, the disposable income of families is relatively low, resulting in people with poor health not only losing productivity and income but also having out-of-pocket (OOP) expenses for necessary healthcare services. Therefore, improved health in developing countries is good for productivity (13–15). Sülkü and Caner (16), Mehrara and Musai (17), and Elmi and Sadeghi (18), and other scholars used cross-sectional interface data to investigate the co-integration relationship between health expenditure and GDP in some developing countries and the causal relationship by adopting the multivariate co-integration method. Both show a strong positive correlation and prove that income is an important factor in health expenditure and growth in many developing countries. Thoa et al. (19) explored the relationship between family healthcare utilization and economic growth in the Bhavi region of Vietnam from 2003 to 2007. Economic growth in developing countries widened the gap between the rich and the poor and also increased the utilization of medical care, and families with economic growth also had better utilization of medical and health services (19). Bedir (20) explored the relationship between medical care and economic growth in developing countries and found that medical care expenditure reached the saturation level, and the impact of medical expenditure on economic growth weakened.

Most of the above studies tend to consider the single relationship between medical and health expenditure and economic development and do not conduct a quantitative assessment of the relationship between medical and economic development. Fortunately, however, the coupled coordination model allows us to study the interaction between two systems (21, 22). In recent years, the coupled coordination model has been applied to a certain extent in China's medical and health field, but it started relatively late. The initial research focused on the coordination relationship between medical input and health benefits (23, 24). After that, various scholars began paying attention to the coupling and coordination of research between medical services and economic development (25–28). The coupling study of traditional Chinese medicine medical treatment and economic development level is subsequent. At present, the coupling coordination model is mainly used to study the regional coupling coordination degrees (29), the coupling coordination degree between China's overall traditional Chinese medicine service ability and regional economy (30), and use spatial analysis methods to explore the spatiotemporal evolution characteristics of coupling coordination degree (31). In general, there are more studies on the causal relationship and co-integration relationship between medical care and social economy and fewer studies on the coupling mechanism of traditional Chinese medicine medical care and social and economic development and the influencing factors of the coupling development of the two systems.

Therefore, the research objective of this article is first to sort out the interaction relationship between traditional Chinese medicine medical services and economic development, then calculate the comprehensive development level of traditional Chinese medicine medical services and economy in 31 provinces of China from 2015 to 2021, couple and coordinate, and explore the influencing factors of coupling and coordination. Finally, policy recommendations are proposed. The contribution of this study is to expand the international empirical research on the coupling of healthcare and economy from a bidirectional perspective and enrich relevant theoretical research. The combination of mathematical statistics and exploratory spatial data analysis can be used to explore the degree of coupling and coordination between economic development and medical services in different countries. Showcasing the coordination status and regional imbalance characteristics between traditional Chinese medicine and the economy is conducive to promoting the sustainable and healthy development of China's medical and health industry and economy.

## 2 The coupling mechanism between traditional Chinese medicine and economic development

### 2.1 Traditional Chinese medicine medical services can effectively promote economic development

The development level of traditional Chinese medicine medical services is one of the important guarantees for economic development. Only from the perspective of medical and healthcare does traditional Chinese medicine have two functions: economy and public health (32). The development of traditional Chinese medicine contributes to the strategy of “Healthy China”, and the direct effect of traditional Chinese medicine on social and economic development can be reflected in the following ways:

(1) Traditional Chinese medicine medical services have spurred the emergence of new industries and provided more employment opportunities. The combination of traditional Chinese medicine services with smart healthcare, artificial intelligence technology, chronic disease treatment, medical and older adults care integration, tourism, and other industries in China has formed emerging industries. Medical institutions provide jobs. It includes the employment of professionals such as traditional Chinese medicine physicians, pharmacists and nurses, as well as employment opportunities in the pharmaceutical industry chain, such as the cultivation, processing and sales of medicinal materials. The development of the traditional Chinese medicine medical industry can create large numbers of employment and entrepreneurial opportunities, contributing to economic growth and employment.

(2) The core purpose of traditional Chinese medicine medical services is to enhance the health stock of residents. Mayer (7) and Mushkin (33) put forward the human capital theory that health, as an important human capital input, is one of the sources of economic growth An increase in the health of the population means an increase in effective working hours and productivity, which in turn affects economic development. Relevant studies have shown that healthy human capital, as an input factor of final product production, can directly promote economic growth (34–36).

(3) The growth of demand for medical services can optimize the economic structure. The demand for health consumption determines consumption expenditure, and the medical consumption expenditure generated by the demand for health services is the most basic livelihood consumption. The provision and demand for medical and health services are usually accompanied by an increase in medical consumption expenditure. The health consumption expenditure of individuals, enterprises, and governments can stimulate the demand of related industries, thereby driving economic growth. The improvement of health levels also increases the demand for health consumption of medical insurance and drives the development of the health service industry. Therefore, the improvement of health levels is conducive to the optimization and upgrading of the economic structure (37, 38). With the improvement of medical and health standards, people's demand for medical services also increases. This promotes the development and expansion of the medical industry and stimulates the growth of medical-related industries. Bhargava et al. (39) found that as the level of public healthcare services improves, it increases the health consumption expenditure of residents, leading to a significant improvement in the economic performance of developing countries.

### 2.2 Economy is the material foundation of traditional Chinese medicine medical services

Social and economic activities also play a decisive role in restricting development levels, service efficiency, and the structure of traditional Chinese medicine medical services. The level of economic development restricts the level of traditional Chinese medicine medical services, and economic development is the foundation of traditional Chinese medicine medical services. It is mainly reflected in the following aspects:

(1) The prosperity of the economy promotes the upgrading of the structure of the traditional Chinese medicine medical industry. Kamanda et al. (5) found that the total economic output affects the input of traditional Chinese medicine medical resources. Generally speaking, the larger the regional economic output, the more investment is made in medical and health service resources. With the prosperity and development of the economy, the government will invest more financial funds into the medical and health service industry, leading to the emergence of more medical and health institutions, which is conducive to expanding the medical service market. In order to improve medical service capabilities, medical service entities will be committed to researching and developing new medical equipment and technologies, promoting innovation and upgrading industrial technology in the medical service industry, and injecting new vitality into the medical service industry.

(2) Economic development affects the allocation of traditional Chinese medicine medical and health resources. The layout of economic development also affects the layout of traditional Chinese medicine medical institutions, the layout structure of traditional Chinese medicine medical services will automatically adapt to the form of economic development and meet the needs of economic development. Economic activities also play an important role in promoting the structural layout, medical service level, and service efficiency of traditional Chinese medicine medical institutions. First, regions with higher levels of economic development attract a large population agglomeration, which affects the demand for medical and health services. Therefore, the layout structure of traditional Chinese medicine medical treatment will automatically adapt to the form of economic development and meet the needs of economic development.

(3) Economic development also affects the local concept of medical treatment, improving health levels. Family capital determines patients' choice of medical treatment. Generally speaking, families in economically developed areas have sufficient family capital and will directly choose medical treatment (40). Residents in economically underdeveloped areas generally choose to delay medical treatment, give up medical treatment or borrow medical treatment, which leads to loss of local human resources, human damage, and curb social and economic development. Personal income is also important for healthcare. Grossman (41) stated that a consumer's demand for health and medical care should be positively correlated with his wage rate. Raghupathi (8) and Tang et al. (37) pointed out that increasing economic income can lead to better nutrition, preventive treatment, good sanitation facilities, and safe drinking water, resulting in high-quality healthcare that improves health.

Overall, traditional Chinese medicine medical services and economic development are not solely promoted, but they have a close mutual promotion effect. The relationship between healthcare and the economy is interdependent and mutually reinforcing. The development of the economy provides strong support and impetus for traditional Chinese medicine, and the progress of traditional Chinese medicine also provides a solid guarantee and driving force for economic development. Therefore, we should strengthen our attention to the integration and interaction between healthcare and the economy, promote their coordinated development, and contribute to high-quality economic development and people's health.

## 3 Research design

### 3.1 Construction of indicator system

#### 3.1.1 Selection of traditional Chinese medicine medical service indicators

The selection of comprehensive evaluation indicators in this study refers to previous relevant studies (25, 42, 43). On the basis of organizing relevant research results, based on the availability, independence and scientificity of the indicators, the comprehensive evaluation index system of this study was determined.

Traditional Chinese medicine medical institutions are one of the main bodies of medical and health services in China. Traditional Chinese medicine medical and health institutions can reflect the distribution density of medical and health services (42), and their quantity and quality may directly affect the future of traditional Chinese medicine services. Traditional Chinese medicine medical institutions, the number of traditional Chinese medicine beds, and traditional Chinese medicine health personnel are the basis of traditional Chinese medicine medical resources, which can directly reflect the supply capacity of traditional Chinese medicine medical services. Financial investment in medical and healthcare can reflect the financial guarantee of medical and healthcare. This research employed the financial allocation of traditional Chinese medicine medical institutions and the revenue and expenditure ratio of traditional Chinese medicine hospitals to reflect the financial guarantee of traditional Chinese medicine medical care. The financial allocation of traditional Chinese medicine medical institutions is an important factor in embodying policy support. The income and expenditure ratio of traditional Chinese medicine hospitals mainly reflects the profit situation of hospitals. This study selected indicators such as the number of diagnosis and treatment visits, discharge visits, per capita daily diagnosis and treatment visits by physicians in traditional Chinese medicine hospitals, per capita daily hospitalization days by physicians in traditional Chinese medicine hospitals, utilization rate of beds in traditional Chinese medicine hospitals, and average hospitalization days in traditional Chinese medicine hospitals to reflect the status of traditional Chinese medicine medical and health services. The average length of stay and the utilization rate of beds are the core indexes to measure the efficiency of ward work, and the average length of stay reflects the ability and efficiency of medical service. The utilization rate of beds is not only an important indicator for measuring the efficiency of wards but also an important indicator for evaluating the management capacity of hospitals and wards. The amount of traditional Chinese medicine consultation and treatment is a key indicator that reflects the utilization of traditional Chinese medicine services and its influencing factors (43). It can also reflect people's trust in traditional Chinese medicine medical services from the side.

#### 3.1.2 Selection economic indicators

Economic aggregate is an important indicator for measuring the development level of a regional economic scale, which can represent the economic strength of a country or region. This study used GDP, local general public budget revenue (100 million yuan), total retail sales of social consumer goods (100 million yuan) and other indicators to measure China's economic aggregate. The economic structure is of great significance to the healthy and sustainable development of the economic system. A reasonable and adaptable economic structure helps to improve the overall efficiency and competitiveness of the economic system and promote the healthy development of the economy. This study uses the proportion of the primary, secondary, and tertiary industries to represent the economic structure. Economic benefit is an index used to the income and results of economic activities. It is represented by per capita consumption expenditure, per capita disposable income (yuan), Engel coefficient, and per capita gross regional product (yuan). The specific indicator system is shown in Table 1.

**Table 1:** Evaluation index system of traditional Chinese medicine and economic development.

**System**	**Target layer**	**Index layer**	**Direction**	**Weight**
Traditional Chinese medicine medical system	Investment in traditional Chinese medicine resources	Traditional Chinese medicine personnel	+	0.106
		Traditional Chinese medicine medical institutions	+	0.118
		Traditional Chinese medicine beds	+	0.093
		Financial allocation for traditional Chinese medicine medical institutions	+	0.106
		Revenue and expenditure ratio of traditional Chinese medicine hospitals	+	0.094
	Volume of traditional Chinese medicine services	Traditional Chinese medicine medical institutions diagnose and treat people/10,000 people	+	0.121
		Number of discharged patients/person from traditional Chinese medicine medical institutions	+	0.106
		Per capita daily diagnosis and treatment by doctors in traditional Chinese medicine hospitals	+	0.123
		Physicians in traditional Chinese medicine hospitals are responsible for daily hospitalization days per person	+	0.050
	Efficiency of traditional Chinese medicine services	Bed usage rate in traditional Chinese medicine hospitals/%	+	0.049
		Average hospitalization days/day in traditional Chinese medicine hospitals	–	0.034
Economic development system	Economic aggregate	Gross regional product	+	0.111
		Local general public budgeting revenue (100 million yuan)	+	0.193
		Total retail sales of social consumer goods/100 million yuan	+	0.120
	Economic structure	Proportion of the primary sector of the economy	–	0.029
		Proportion of the secondary sector of the economy	+	0.029
		Proportion of the tertiary sector of the economy	+	0.122
	Economic benefits	Per capita consumption expenditure by region	+	0.122
		Per capita disposable income (yuan)	+	0.138
		Engel's coefficient	–	0.033
		Per capita gross regional product (yuan)	+	0.104

The data in this study are a panel data set, and the weight of indicators in the table is the average weight value from 2015 to 2021.

### 3.2 Research methods

The research topic of this article is to measure the comprehensive development level, coupling coordination degree, and influencing factors of traditional Chinese medicine medical services and economic development in 31 provinces of China from 2015 to 2021. The relevant indicator data of traditional Chinese medicine medical services and economy in 31 provinces of China from 2015 to 2021 were used as research samples. China's 31 provinces are divided into four major regions, namely the eastern, central, western, and northeastern regions, as the main research and analysis areas. Therefore, relevant variables were selected from the two systems of traditional Chinese medicine services and economic development to calculate their comprehensive development level, and a coupling coordination model was used to calculate their coupling coordination scheduling. The Tobit model was used to calculate the factors influencing the coupling coordination degree in the four major regions. The research object of this article is 31 provinces in China. The data sources for this study were all from the China Statistical Yearbook, China Health Statistical Yearbook, and Statistical Extracts of Chinese Traditional Medicine from 2016 to 2022. Hong Kong, Macau, and Taiwan Provinces were not included in the analysis due to their inconvenient data collection.

(1) Entropy method

The size of indicator data was different, and there were differences in positive and negative orientations. The *Min-Max* method was adopted to standardize the data to ensure that the value of all indicator data was between 0 and 1. *i* represents the province and *j* represents the indicator, and the formula is as follows:


$$\begin{array}{l} \text{Positive indicator}:\;{X^{\prime }}_{ij}\;=\frac{X_{ij}\;-Min_{ij}}{Max_{ij}\;-Min_{ij}}\\ \text{Negative indicator}:\;{X^{\prime }}_{ij}\;=\frac{Max_{ij}\;-X_{ij}}{Max_{ij}\;-Min_{ij}} \end{array}$$

where *Min*_*ij*_ and *Max*_*ij*_ represent the minimum and maximum value of *j* index in province *i*, respectively, and *X*_*ij*_ represents the value of *j* index in province *i*. ${X^{\prime }}_{ij}$ is the normalized value. *i* = 1,2,3... *m, j* = 1,2,3... *n*.

First of all, the weight of each indicator is measured. The formula is as follows:


$$P_{ij}\;=\;\frac{{X^{\prime }}_{ij}}{\sum_{i=1}^{m}{X^{\prime }}_{ij}}$$

where *P*_*ij*_ is the proportion value of *j* indicators in the *i* region. Then the information entropy value *e*_*j*_. The formula for information entropy is as follows:


$$e_{j}\;=\;-k\sum_{i=1}^{m}P_{ij}Ln(P_{ij}+0.0001),\;0\;\leq e_{j}\;\leq \;1$$

where $k=\frac{1}{\ln_{m}}$. Finally, the indicator weights of the traditional Chinese medicine and economic development system are calculated.


$$\begin{array}{l} w_{j}\;=\;\frac{\left(1-e_{j}\right)}{\sum_{j=1}^{n}\left(1-e_{j}\right)} \end{array}$$

where *w*_*j*_∈[0,1], and the sum of all terms is 1, $\overset{n}{\underset{j=1}{\sum }}w_{j}=\;1$.

The standard values and weights of each indicator are obtained to calculate the comprehensive evaluation value *U* of each system *U*_1_, *U*_2_. Comprehensive evaluation function. The formula is as follows:


$$\begin{array}{l} U_{i=1,2}=\sum_{j=1}^{n}{X^{\prime }}_{ij}W_{j} \end{array}$$

where *U*_1_ is the comprehensive evaluation value of traditional Chinese medicine, *U*_2_ is the comprehensive evaluation value of economic development. ${X^{\prime }}_{ij}$ is the standardized number, and ${X^{\prime }}_{ij}$ is the indicator weight.

(2) Coupling coordination model

The coupling degree refers to the interaction and influence between two or more systems, reflecting the degree of interdependence and constraints between systems (31, 44). This study uses a coupled coordination model to calculate the coupled coordination scheduling between traditional Chinese medicine and social economy. The coupling formula for the two systems is as follows:


$$\begin{array}{l} C=2*{\left\{{U_{1}U_{2}/\left(U_{1}+U_{2}\right)}^{2}\right\}}^{1/2} \end{array}$$

where *C* is the coupling degree between the two, where *C*∈[0,1]. Relying solely on coupling degree discrimination may be misleading, and a coupling coordination degree model needs to be established, namely:


$$\begin{array}{l} T=aU_{1}+\beta U_{2}\\ \;D=\sqrt{C*T} \end{array}$$

where *D* is coupling co-scheduling, *T* is coordination index, α, β is an undetermined coefficient that satisfies α+β=*1—a*ssuming that both contribute equally to the coordinated development of coupling, i.e., α = β = 0.5. The level division of coordination degree between the two systems is shown in Table 2.

**Table 2:** Hierarchy of coupling coordination between traditional Chinese medicine and economic development.

**Coordination level**	***D*-value**	**Coupling development type**
Low-level coordination (antagonistic period)	(0–0.1)	Extreme dysregulation and decline
	(0.1–0.2)	Severe dysregulation and decline
	(0.2–0.3)	Moderate dysfunctional decline
	(0.3–0.4)	Minor disorder
Middle-level coordination (break-in period)	(0.4–0.5)	Proximity coordination (transitional)
	(0.5–0.6)	Barely coordinated (transitional)
	(0.6–0.7)	Primary coordination
High-level coordination (coordination period)	(0.7–0.8)	Intermediate coordination
	(0.8–0.9)	Well-coordination
	(0.9–1)	High-quality coordination

(3) Tobit panel model

Due to the coupling coordination degree being between 0 and 1 and being a restricted dependent variable, a panel Tobit model can be used to analyze the influencing factors of the coordination degree between the two systems. To avoid multicollinearity and heteroscedasticity, logarithmic treatment is applied to all explanatory variables. The model formula is constructed as follows:


$$\begin{array}{l} D_{it}\;=y_{0}\;+y_{1}\;lnTcmp_{it}+\;y_{2}lnGov_{it}\;+\;y_{3}lnPop_{it}\;+y_{4}lnLed_{it}\\ \;+\;y_{5}lnPdr_{it}+y_{6}lnTrain_{it}+y_{7}lnIndu_{it}+\varepsilon_{i} \end{array}$$

In the formula, *D*_*it*_ is coupled co-scheduling; *y*_0_ is a constant term; Tcmp represents human capital in traditional Chinese medicine, while gov represents government financial capacity in healthcare; Pop represents population density, Led represents economic development level, Pdr represents population mortality rate, Train represents transportation infrastructure, and Indu represents industrial structure. I represents each province, and t represents time; it is a random perturbation term.

## 4 Research results and analysis

### 4.1 The comprehensive development level of traditional Chinese medicine medical services and social economy

The comprehensive development coefficient of traditional Chinese medicine (U1), and the comprehensive development coefficient of social and economic (U2) were arithmetically averaged, respectively, so as to indicate the average development level of the two systems in each region during 2015–2021. In addition, based on U1/U2 = F, the relative development degree of the two systems is calculated. An *F*-value of <0.8 indicates that the development of traditional Chinese medicine is lagging behind the level of economic development. Notably, 0.8 <F <1.2 indicates that the two systems are developing synchronously. If F > 1.2, it indicates that the level of traditional Chinese medicine is leading the development of economic development. China's 31 provinces are divided into four regions (eastern, central, western, and northeastern) for analysis.

As shown in Table 3, The u1 in the eastern region increased from 0.409 in 2015 to 0.392 in 2021, indicating that the traditional Chinese medicine medical level in the eastern region of China has slightly decreased in recent years, but U2 rose from 0.312 to 0.486, denoting that the social and economic development level in the eastern region has greatly improved from 2015 to 2021. In 2021, U1 and U2 were 0.392 and 0.486, respectively, indicating that the social and economic development was ahead of the development level of traditional Chinese medicine. The *F*-value decreased from 1.355 to 0.890, demonstrating that the gap between the two systems gradually narrowed and synchronous development was achieved. The central U1 increased from 0.367 to 0.373, indicating that Chinese medicine in the central region was seeking progress while maintaining stability. The rise of U2 from 0.156 to 0.270 indicates that the level of social and economic development in the central region has risen sharply, and the level of traditional Chinese medicine medical treatment is higher than the level of economic development. The *F*-value decreased from 2.348 in 2015 to 1.373 in 2021, which reveals that the gap between the two systems is narrowing and gradually tends to be synchronized. U1 in the western region changed from 0.326 to 0.328, which denotes that the level of traditional Chinese medicine in the west has not been greatly improved and has developed steadily. U2 increased from 0118 to 0.175, which indicates that the social economy of the western region has improved to a certain extent in recent years, and the level of traditional Chinese medicine is higher than the level of social and economic development. The F-value also changed from 2.852 in 2015 to 1.919 in 2021, which demonstrates that the gap between the medical level and the level of social and economic development in the western region is gradually narrowing and tends to develop simultaneously. In Northeast China, U1 changed from 0.225 to 0.184, indicating that the level of traditional Chinese medicine in Northeast China is gradually decreasing, and U2 changed from 0.306 to 0.177, denoting that the social economy in Northeast China has not improved in recent seven years, but has declined rapidly. The *F*-value changed from 1.014 in 2015 to 1.148 in 2021, indicating that the development gap between traditional Chinese medicine and social economy in Northeast China has gradually widened.

**Table 3:** Comprehensive development level degree of traditional Chinese medicine and social economy in China.

	**Year**	**2015**	**2021**	**2015**	**2021**	**2015**	**2021**
	**Province**	**U1**	**U1**	**U2**	**U2**	**F**	
Eastern	Beijing	0.346	0.263	0.462	0.684	0.749	0.384
	Tianjin	0.221	0.145	0.302	0.363	0.733	0.401
	Hebei	0.383	0.431	0.164	0.247	2.339	1.748
	Shandong	0.524	0.554	0.308	0.449	1.700	1.233
	Jiangsu	0.544	0.489	0.388	0.653	1.402	0.748
	Zhejiang	0.579	0.526	0.347	0.584	1.669	0.900
	Shanghai	0.400	0.353	0.441	0.708	0.908	0.498
	Fujian	0.318	0.320	0.219	0.381	1.451	0.841
	Guangdong	0.691	0.695	0.389	0.655	1.777	1.061
	Hainan	0.084	0.148	0.102	0.137	0.823	1.081
	Eastern mean	0.409	0.392	0.312	0.486	1.355	0.890
Northeast China	Liaoning	0.240	0.166	0.231	0.252	1.038	0.659
	Jilin	0.204	0.207	0.544	0.157	0.376	1.315
	Heilongjiang	0.231	0.179	0.142	0.122	1.629	1.470
	Northeast average	0.225	0.184	0.306	0.177	1.014	1.148
Central section	Shanxi	0.212	0.222	0.150	0.199	1.412	1.117
	Anhui	0.326	0.373	0.132	0.289	2.465	1.292
	Jiangxi	0.321	0.316	0.118	0.225	2.716	1.406
	Henan	0.492	0.554	0.173	0.293	2.840	1.892
	Hubei	0.421	0.370	0.191	0.320	2.204	1.158
	Hunan	0.427	0.401	0.174	0.291	2.453	1.377
	Middle mean	0.367	0.373	0.156	0.270	2.348	1.374
West	Inner Mongolia	0.270	0.243	0.187	0.224	1.444	1.086
	Guangxi	0.372	0.404	0.100	0.150	3.708	2.696
	Chongqing	0.365	0.376	0.170	0.274	2.145	1.373
	Sichuan	0.734	0.733	0.165	0.297	4.439	2.469
	Guizhou	0.295	0.338	0.083	0.150	3.544	2.261
	Yunnan	0.382	0.390	0.098	0.177	3.900	2.204
	Tibet	0.193	0.164	0.074	0.097	2.606	1.687
	Shaanxi	0.340	0.307	0.142	0.227	2.395	1.351
	Gansu	0.320	0.308	0.085	0.108	3.762	2.848
	Qinghai	0.167	0.208	0.094	0.114	1.774	1.822
	Ningxia	0.158	0.204	0.112	0.145	1.414	1.408
	Xinjiang	0.317	0.257	0.102	0.141	3.094	1.820
	Western mean	0.326	0.328	0.118	0.175	2.852	1.919
	National average	0.351	0.343	0.206	0.294	2.094	1.407

As shown in Table 3, in 2021, the average value of U1 and U2 in the overall region of China was 0.343 and 0.294, respectively, which denotes that the level of traditional Chinese medicine medical treatment is generally higher than the level of social and economic development. The F-value changes from 2.094 to 1.407, which indicates that the development gap between the two systems in China is gradually decreasing, and the development balance of the two systems is rising and tends to be stable on the whole. It can be seen that in recent years, China has focused on coordinated development strategy has achieved certain results in medical and economic aspects. With the exception of Northeast China, both the level of traditional Chinese medicine medical services and the level of social and economic development in China decrease from east to west, and the level of social and economic development in Northeast China is not much different from that in western China. The development level of traditional Chinese medicine and social and economic development is developing simultaneously only in the eastern region, and the development level of the two systems is gradually increasing from the east to the west, but the development gap between the two systems in the central and western regions tends to develop simultaneously. In general, the levels of medical development in China are higher than the level of economic development, and the development level of the two systems presents the distribution characteristics of “the highest in the east, followed by the middle and west, and the lowest in the northeast.” Yang Huan's study also obtained similar results (45).

### 4.2 The coupling and coordination characteristics of traditional Chinese medicine medical services and economy

#### 4.2.1 Time series evolution of coupling coordination

As can be seen from Table 4, in 2015, there were five provinces with mild imbalances (0.3–0.4), 12 on the verge of coordination (0.4–0.5), eight barely coordinated (0.5–0.6), five primarily coordinated (0.6–0.7), and 1 with intermediate coordination (0.7–0.8). It can be seen that the coordination degree of China's two systems in 2015 was mainly near coordination and barely coordination, which belongs to the middle level of coordination (run-in period). The stability of middle-level coordination is not strong and belongs to the transitional type. If the force point inside the two systems can be found, the coupling coordination will rise to the coupling period; on the contrary, it is easy to fall to the low -level coordination (antagonistic period). In 2021, there were four mildly dysfunctional provinces, 10 on the verge of coordination, nine barely coordinated, three with primary coordination, four with intermediate coordination, and one well-coordinated. The low-level coordination provinces decreased, and the middle and high-level coordination provinces increased, which shows that the overall coordination level gradually shifted upward.

**Table 4:** Coupling coordination degree of Traditional Chinese Medicine and Economy in China from 2015 to 2021.

**Region**	**Province/city**	**2015**	**2016**	**2017**	**2018**	**2019**	**2020**	**2021**	**Rising rate**
Eastern	Beijing	0.632	0.674	0.677	0.701	0.704	0.623	0.651	0.02%
	Tianjin	0.508	0.540	0.522	0.481	0.544	0.458	0.479	−0.03%
	Hebei	0.500	0.550	0.561	0.539	0.596	0.584	0.571	0.07%
	Shandong	0.634	0.695	0.699	0.660	0.724	0.702	0.706	0.07%
	Jiangsu	0.678	0.739	0.744	0.698	0.750	0.745	0.752	0.07%
	Zhejiang	0.670	0.720	0.727	0.688	0.746	0.737	0.744	0.07%
	Shanghai	0.648	0.689	0.694	0.648	0.642	0.684	0.707	0.06%
	Fujian	0.514	0.556	0.554	0.529	0.583	0.577	0.591	0.08%
	Guangdong	0.720	0.802	0.808	0.742	0.814	0.821	0.822	0.10%
	Hainan	0.304	0.338	0.322	0.311	0.398	0.407	0.378	0.07%
	Eastern mean	0.581	0.630	0.631	0.600	0.650	0.634	0.640	0.06%
The Northeast	Liaoning	0.485	0.502	0.501	0.456	0.539	0.469	0.452	−0.03%
	Jilin	0.577	0.438	0.459	0.422	0.479	0.420	0.425	−0.15%
	Heilongjiang	0.425	0.446	0.443	0.414	0.466	0.373	0.384	−0.04%
	Northeast average	0.496	0.462	0.468	0.431	0.495	0.421	0.420	−0.08%
Central region	Shanxi	0.423	0.462	0.447	0.432	0.530	0.460	0.459	0.04%
	Anhui	0.455	0.516	0.523	0.495	0.561	0.566	0.573	0.12%
	Jiangxi	0.441	0.483	0.477	0.455	0.509	0.510	0.516	0.08%
	Henan	0.540	0.594	0.600	0.576	0.657	0.634	0.635	0.10%
	Hubei	0.532	0.574	0.578	0.547	0.624	0.571	0.586	0.05%
	Hunan	0.522	0.567	0.571	0.542	0.611	0.589	0.584	0.06%
	Middle mean	0.486	0.533	0.533	0.508	0.582	0.555	0.559	0.07%
West	Inner Mongolia	0.474	0.504	0.508	0.475	0.514	0.480	0.483	0.01%
	Guangxi	0.439	0.606	0.478	0.458	0.501	0.492	0.496	0.06%
	Chongqing	0.499	0.531	0.538	0.506	0.568	0.556	0.567	0.07%
	Sichuan	0.590	0.649	0.662	0.621	0.702	0.680	0.683	0.09%
	Guizhou	0.396	0.427	0.437	0.422	0.468	0.458	0.474	0.08%
	Yunnan	0.440	0.475	0.471	0.448	0.520	0.514	0.512	0.07%
	Tibet	0.345	0.320	0.341	0.359	0.365	0.332	0.356	0.01%
	Shaanxi	0.469	0.504	0.508	0.485	0.517	0.498	0.514	0.05%
	Gansu	0.406	0.435	0.433	0.414	0.459	0.441	0.427	0.02%
	Qinghai	0.355	0.384	0.402	0.383	0.411	0.404	0.393	0.04%
	Ningxia	0.364	0.400	0.462	0.434	0.428	0.387	0.415	0.05%
	Xinjiang	0.424	0.441	0.453	0.420	0.484	0.405	0.436	0.01%
	Western mean	0.433	0.473	0.474	0.452	0.495	0.471	0.480	0.05%
	Nationwide	0.497	0.534	0.536	0.508	0.562	0.535	0.541	0.04%

As can be seen from Table 4, from 2015 to 2021, the coordination degree between traditional Chinese medicine medical treatment and social economy in China increased from 0.497 to 0.541. The two systems in China have risen from near imbalance to barely coordinated, and the average rate of increase of the degree of coordination between the two systems was 0.04%. Eastern rose from 0.581 to 0.640, an increase of 0.06%. The central region increased by 0.559 from 0.486, an increase rate of 0.07%. The west rose from 0.433 to 0.480, an increase of 0.05%. The northeast decreased from 0.496 to 0.420, with an increase of −0.08%. The reason for the decline of coordination degrees in Northeast China may be that there are only three provinces in Northeast China, which are mainly dominated by the secondary industry, a remote location with a low population density. It can be seen that the development rate of coupling coordination degree in central and eastern regions was the fastest, and the western region was also catching up. However, the reason why China's overall development speed is relatively slow is that the coupling and coordination degrees of the two systems in the northeast region is declining, and the decline speed is evident.

**Figure 1:**
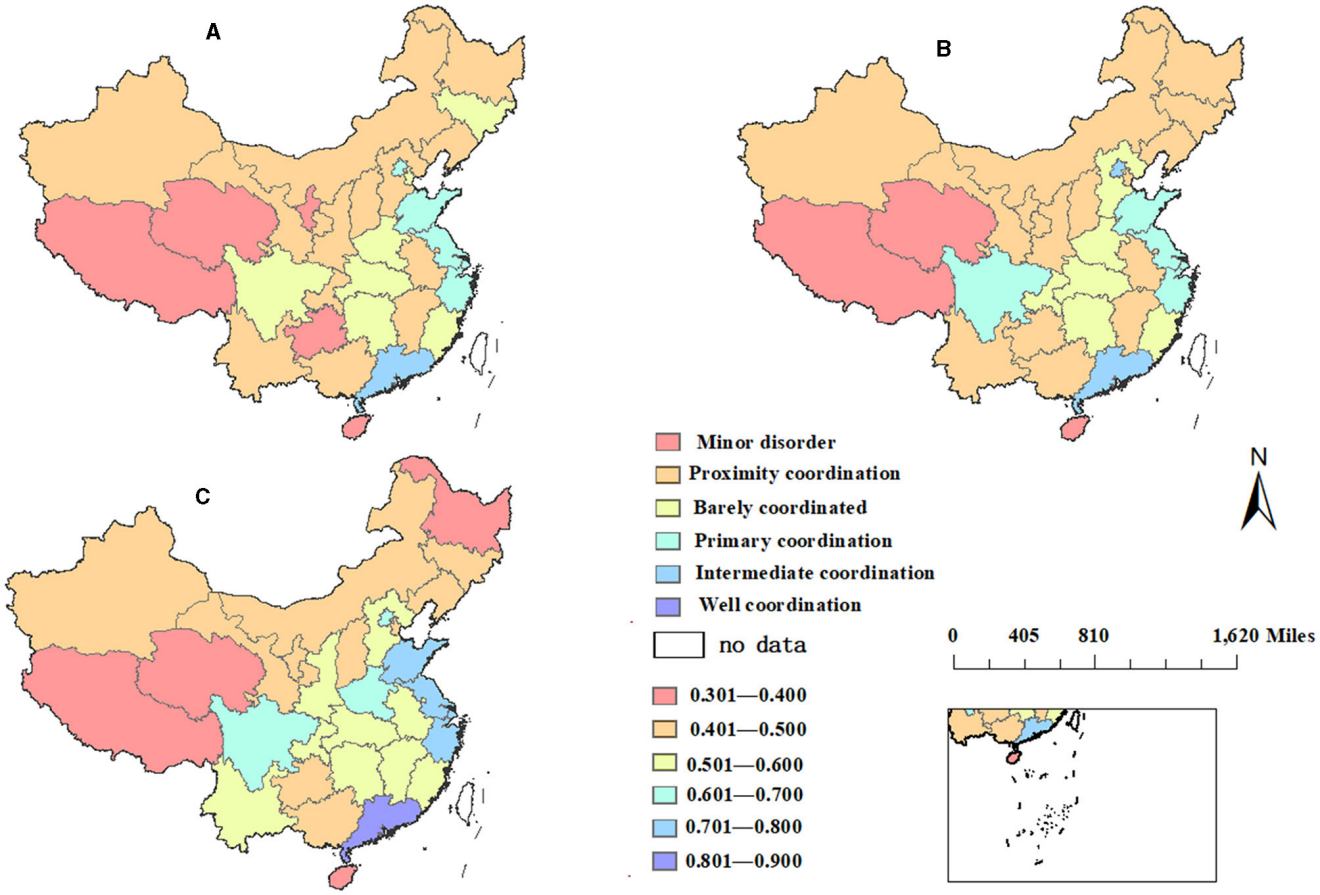
Spatial comparison of coupling coordination degree in **(A)** 2015, **(B)** 2018, and **(C)** 2021.

China is a vast region. In order to more directly show the spatial distribution characteristics of the coupling coordination degree of various provinces and regions in China, the ArcGis10.8 software was used in this study to visually compare and analyze the coupling coordination degrees of two systems in various regions of China in 2015, 2018, and 2021, as shown in Figure 1.

This is shown in Figure 1A; in 2015, the provinces with youth imbalance were scattered mainly in the western provinces of Tibet, Qinghai and Guizhou, and the eastern provinces of Hainan. The provinces at risk of coordination were scattered across China: Xinjiang, Gansu, Inner Mongolia, Heilongjiang, Liaoning, Shaanxi, Shanxi, Hebei, Yunnan, Chongqing, Guangxi, Jiangxi, and Anhui. The provinces that were barely coordinated were mainly in the central region, namely Sichuan, Hubei, Henan, Fujian, and Jilin. The primary coordination provinces were Beijing, Shandong, Jiangsu, and Zhejiang. The province with the best degree of coordination was Guangdong Province, which had intermediate coordination in 2015. Guangdong Province, the province with the highest degree of coordination, has been far ahead. This is mainly due to the superior geographical location of Guangdong Province and the good mass base of traditional Chinese medicine (46).

This is shown in Figure 1B; after 3 years of development, the coupling coordination degrees of various regions in China changed to a certain extent in 2018. In Ningxia, Sichuan, Guizhou, and Chongqing, the coupling coordination degree of these provinces has increased by one level. As for the coastal areas, the changes are less, and Jilin in northeast China has decreased from barely coordinated to nearly coordinated. In other provinces, there was no significant change.

This is shown in Figure 1C; compared with 2015, the coupling degree of each province in 2021 was more perceivable. The eastern coastal areas, such as Shandong, Jiangsu, Zhejiang, and other areas, have been greatly developed from barely coordinated to primary coordination. Because these provinces have better economic foundation development, higher per capita income, higher health literacy than the central and western regions, and wide coverage of transportation infrastructure, the overall accessibility of medical and health resources is high. High accessibility of medical and health resources. The central and western provinces of Shaanxi, Yunnan, Jiangxi, and Anhui have also progressed from near-imbalance to barely coordinated. These provinces have a strong endowment of traditional Chinese medicine resources and a certain mass base; thus, the level of medicine development is relatively high. The coupling coordination degrees of Hainan, Xizang, and Qinghai provinces have always been slightly disordered. These three provinces are located in the periphery of China, especially in the western region, where the economic foundation of Tibet and Qinghai provinces is weak, and it is difficult to improve the coupling coordination degree. The coordination degree in Heilongjiang has significantly decreased, from near coordination to mild imbalance, which may be due to the low population density of the province in the periphery of China.

In summary, the spatial distribution characteristics of the coupling coordination degree showed a stepped development, with east > central > west > northeast. The overall coordination degree was consistent with the spatial distribution of China's economic development level, as well as with China's population line—“Hu Huanyong line”, which indicates that economic development is the basis for the coordinated development of the two systems. On the whole, the economy and population have a strong influence, among which economic factors are the basic factors because economy and population will directly affect the distribution structure of medical resources.

#### 4.2.2 Spatial autocorrelation analysis of coupling coordination degree

Based on the coupling coordination degree of relevant data, the ArcGIS software was used to measure the global autocorrelation of China and calculate the global Moran's I index of traditional Chinese medicine and economic development in 31 provinces of China from 2015 to 2021. The larger the value of Global Moran's I, the stronger the spatial agglomeration; the smaller the value, the stronger the spatial dispersion (21, 47). According to Table 5, the global Moran index of the coupling coordination degree between traditional Chinese medicine medical treatment and social and economic development is positive, and the value is expanding. In addition to the 95% significance of the global Moreland index in 2015 (*p* < 0.05), the significance test of the global Moreland index in other years was 99% (*p* < 0.01), indicating that there is a strong spatial agglomeration relationship between the coupling and coordination of traditional Chinese medicine medical treatment and social economy. The global Moran index increases gradually over time, indicating that the spatial agglomeration of the coupling coordination degree of the two systems is becoming stronger and stronger. The coupling and coordination between traditional Chinese medicine medical services and the regional economy have a strong spatial dependence on the surrounding regions. That is, if a province has a high value, the coupling and coordination degree of its neighboring provinces is also relatively high, and regional clustering is more obvious. We should pay attention to the spatial pattern of the coupling development of traditional Chinese medicine medical services and regional economy, follow the development laws of medical services and economy, encourage the construction of collaborative development mechanisms between adjacent regions, and establish cross-regional cooperation and multi-center professional cooperative alliances.

**Table 5:** Spatial autocorrelation table of coupling coordination degree between two systems in China.

**Year**	**Global Moran's I**	***Z***	***P***	**Confidence level**
2015	0.240485	2.324338	0.020107	0.950
2016	0.32513	3.049738	0.00229	0.990
2017	0.272945	2.613012	0.008975	0.990
2018	0.277691	2.642034	0.008241	0.990
2019	0.262437	2.512711	0.011981	0.950
2020	0.378086	3.496081	0.000472	0.990
2021	0.388772	3.579752	0.000344	0.990

### 4.3 Analysis of the influencing factors of coupling co-scheduling

#### 4.3.1 Variable selection

There are multiple factors that affect the coordinated development of traditional Chinese medicine medical services and the economy. Based on existing research and the availability of comprehensive indicator data, combined with the actual situation in China, there are many influencing factors driving the coordinated development of the two systems. With reference to the existing studies combined with the current situation in China, this study mainly analyzes the factors influencing the two systems, from the aspects of medical personnel, medical financial expenditure, economic base, population density, health level, transportation, and industry. Finally, factors such as traditional Chinese medicine human capital (Tcmp), government medical expenditure capacity (gov), population distribution density (Pop), economic development water (Led), population health (Pdr), transportation infrastructure level (Train), and industrial structure (Indu) were determined as explanatory variables. The coupling coordination degree of 31 provinces in China from 2015 to 2021 is used as the dependent variable (see Table 6 for details). The relevant data for all explanatory variables are sourced from the China Statistical Yearbook, China Health Statistics Yearbook, and China Traditional Chinese Medicine Statistics Excerpt. Some data are obtained through secondary calculations.

**Table 6:** Factors affecting coupled co-scheduling.

	**Variable name**	**Variable symbol**	**Variable description**	**Unit**
Explained variable	Coupling coordination degree	D	Calculation results of the coupling coordination model between two systems	
Explanatory variable	Traditional Chinese medicine human capital	Tcmp	Traditional Chinese medicine personnel per thousand people = number of traditional Chinese medicine personnel at the end of the year/population ^*^ 1,000	
	Government's medical expenditure capacity	Gov	Proportion of financial expenditure on traditional Chinese medicine in healthcare expenditure	%
	Population distribution	Pop	Population density = year-end population/provincial area	%
	Economic development level	Led	Per capita disposable income	Yuan/person
	Population health	Pdr	Population mortality rate = number of deaths/year-end population	%
	Transportation infrastructure level	Train	Highway density = highway mileage/provincial area	km/km^2^
	Industrial structure	Indu	Value added of tertiary sector of the economy/secondary sector of the economy	%

#### 4.3.2 Analysis of regression results

Tcmp represents traditional Chinese medicine personnel per thousand people and represents the human capital of traditional Chinese medicine healthcare. According to Table 7, the determinability coefficient of Tcmp for the integrated development of traditional Chinese medicine and social economy in China is −0.0422, which did not pass the significance test, indicating that human capital in traditional Chinese medicine has a certain negative effect on the two systems. This may be the overall growth of the number of traditional Chinese medicine talents, but the allocation of traditional Chinese medicine talent resources was unbalanced. In some regions, the supply exceeds the demand, while in some regions, the supply is less than the demand, which leads to the inadequacy of medical human capital to promote the integrated development of the two systems. The determinability coefficient of traditional Chinese medicine medical manpower for the eastern region is −0.111. A significance test of 5% indicates that traditional Chinese medicine medical manpower capital has a significant negative effect on the coupling coordination between the two systems in the eastern region. The reason may be that the total amount of traditional Chinese medicine medical human resources in the east is large, but the demand for medical services is insufficient, resulting in the unreasonable supply and demand allocation of the total amount of medical personnel. The determinability coefficient of Northeast China is 0.129, which did not pass the significance test, indicating that traditional Chinese medicine manpower in Northeast China has a non-significant promoting effect on the coordinated development of the two systems. The total number of traditional Chinese medicine medical personnel in the northeast is insufficient, the aging is serious, and the total number of medical personnel is less than the medical demand, resulting in the human capital of traditional Chinese medicine has a certain negative effect on the coupling degree of the two systems. Traditional Chinese medicine medical human resources have a positive promoting effect on the central and western regions, with determinable coefficients of 0.0182 and 0.0146, respectively, but have not passed the significance test. It shows that there is still a large shortage of traditional Chinese medicine medical personnel in the central and western regions, and the medical personnel allocation structure is unreasonable. The regional government should also strengthen the investment and training of traditional Chinese medicine human resources.

**Table 7:** Tobit panel regression model results by region in China.

**Dependent variable**	**Nationwide**	**Eastern region**	**Central region**	**Western region**	**Northeast region**
Tcmp	−0.0422	−0.111^**^	0.0182	0.0146	0.129
	(0.031)	(0.055)	(0.045)	(0.053)	(0.114)
Gow	0.0109^***^	0.0070	0.0426^***^	0.00800^*^	0.0444^***^
	(0.003)	(0.005)	(0.012)	(0.004)	(0.015)
Pop	0.0329^***^	0.0231	0.176^***^	0.0375^***^	0.624^**^
	(0.012)	(0.056)	(0.061)	(0.013)	(0.256)
Led	0.141^***^	0.163^***^	0.103^**^	0.0829	0.0961
	(0.030)	(0.055)	(0.041)	(0.055)	(0.148)
Train	0.00812	0.148^**^	0.00722	0.00434	−0.881^**^
	(0.012)	(0.063)	(0.060)	(0.011)	(0.377)
Pdr	−0.107^***^	0.00550	−0.0129	−0.0981^**^	−0.0357
	(0.029)	(0.073)	(0.059)	(0.041)	(0.177)
Indu	0.00685	0.0110^*^	0.0171	0.00186	0.00185
	(0.004)	(0.006)	(0.035)	(0.026)	(0.006)
_cons	−0.870^***^	−1.343^**^	−1.329^**^	−0.291	−3.826^***^
	(0.309)	(0.569)	(0.579)	(0.551)	(1.304)
*N*	217	70	42	84	21
*R* ^2^	0.940	0.985	0.942	0.937	0.996

Standard errors in parentheses. Significance test ^*^p < 0.1, ^**^p < 0.05, ^***^p < 0.01.

*Gov* represents the government's financial expenditure on traditional Chinese medicine and is representative of the government's financial capacity. The regression coefficient of China's medical financial expenditure capacity was 0.0109, which passed the significance level test of 1%, indicating that the medical financial expenditure of traditional Chinese medicine has a positive effect on promoting the coordinated development of the two systems. Under normal circumstances, the more sufficient government financial funds are, the more investment there is in medical infrastructure and human capital cultivation. As can be seen from Table 7, the financial capacity of traditional Chinese medicine medical services has a significant promoting effect on the coordinated development of the two systems in the central, western, and northeastern regions, with determination coefficients of 0.0426, 0.008, and 0.044, respectively, and passing significance tests of 5, 10, and 1%, respectively, which reflects that China's medical financial investment is more inclined to the central and western regions. The effect of medical finance on the coordinated development of the east and northeast regions is not pronounced, indicating that the eastern has not played an obvious role in obtaining traditional Chinese medicine medical finance. The eastern region should consider reasonable planning of medical financial investment to improve the effectiveness of resource allocation.

*Pop* stands for population density. According to Table 7, for the whole country, its regression coefficient was 0.0329, and through the 1% significance test, it indicates that densely populated places have a significant positive effect on the integration and development of the two systems. The positive impact of population on the coupling coordination degree of the eastern and northeastern regions is not strong, with determination coefficients of 0.0231 and 0.0231, respectively, which did not pass the significance test. The population density in the eastern region is too dense, which may also cause a surplus of labor force, increase the medical burden, and bring greater pressure on social and economic development. Population density has no evident positive effect on Northeast China, which is mainly affected by the serious population loss and low fertility rate. The regression coefficients of population density in the central and western regions were 0.176 and 0.0375, respectively, and both passed the significance test of 1%, indicating that population density has a relatively significant effect on the coordinated development of the two systems in the central and western regions.

*Led* represents per capita disposable income, a measure of economic level. According to Table 7 for the whole region of China, the regression coefficient of per capita disposable income was 0.141 and passed the significance test of 1%, which indicates that the level of economic development is the most important factor in promoting the coordinated development of the two systems. For the whole country, the economic level plays a positive role in the coordinated development of the two systems. Research shows that the higher the level of economic development, the greater the total investment in medical resources will be, thus effectively having healthy human capital and promoting social and economic development. As can be seen from the table, the coefficients of the eastern and central regions were 0.163 and 0.103, respectively. The tests of 1 and 5% indicate that the social and economic development levels of the eastern and central regions are relatively high, and traditional Chinese medicine is adapted to the current level of economic development and can promote the coordinated development of the two systems. The regression coefficients for the western and northeastern regions are 0.0829 and 0.0961, respectively, which did not pass the significance test, indicating that the economic development promotion effect of the western and northeastern regions is not significant, and the level of social and economic development needs to be improved.

*Train* stands for highway mileage and is a manifestation of traffic accessibility. On the one hand, the increase in traffic network density can provide convenient conditions for the flow of medical resources and residents' medical treatment. According to Table 7, in the whole country, the coefficient of determination of transportation infrastructure was 0.00812, which failed the significance test, and the level of transportation infrastructure construction has no positive effect on the promotion of the two systems. The coefficient of determination of the eastern regions was 0.148, and it passes the significance test of 5%, indicating that it has a promoting effect on the integration and development of the two systems in the eastern regions. In the eastern regions, the terrain is relatively flat, the traffic density is relatively high, the population density is comparatively high, and the relative availability of medical resources is also relatively high. However, the promotion effect of Train in the central and western regions is not obvious; the determinable coefficients of highway density in the central and western regions are 0.00722 and 0.00434. The western region is sparsely populated, the terrain is large, the economic foundation is weak, its road density is low, and the accessibility of medical resources is poor. The determinable coefficient of highway density in Northeast China is −0.881, and through a significance test of 5%, it indicates that the transportation infrastructure in Northeast China has a significant inhibitory effect on the coordinated development of the two systems. This is mainly due to the vast territory and sparse population in Northeast China, where the overall highway passenger transportation in the three provinces of Northeast China is in a weakly connected state. The Chinese government also needs to strengthen the construction of transportation infrastructure in the central and western regions and do a good job of spatial planning of medical service institutions to provide a more convenient medical treatment pattern for the masses.

*Pdr* population mortality is a negative indicator. The representative of health, in a certain number of years, the population mortality rate has decreased steadily, indicating that the living standards of the region have gradually improved, and the health level has been promoted. According to Table 7, from the perspective of the whole country, the mortality rate has a certain negative effect on the coordination degree of the two systems; the determinable coefficient of population mortality rate is −0.107 and has passed the significance test of 1%, indicating that the overall health level of China needs to be improved. This negative impact is not significant in the central and northeastern regions but is more pronounced in the western regions. The determinability coefficient for the eastern region is 0.0055, which did not pass the significance test, indicating a relatively high level of healthy development in the eastern region. However, the determinability coefficients for the central and northeastern regions were −0.0129 and −0.0357, both of which did not pass the significance test, indicating that the health level of the central and northeastern regions has an insignificant inhibitory effect on the coordinated development of the two systems. However, the determinability coefficient in the western region is −0.0981, and through a 10% significance test, it indicates that the health level in the western region has a significant inhibitory effect on the coordinated development of the two systems. The central, northeastern, and western regions should focus on improving their health levels and enhancing the fairness and accessibility of medical resources.

*Indu* industrial structure, the optimization of industrial structure plays an important role in promoting traditional Chinese medicine medical services and economic development. According to Table 7, the determination coefficients of industrial structure for China, central, western, and northeastern regions are 0.0085, 0.0171, 0.00186, and 0.00185, respectively, all of which have not passed the significance test. This indicates that the industrial structure has no significant promoting effect on the overall coupling and coordination between traditional Chinese medicine medical services and the economy in China. The determinability coefficient of the eastern region is 0.011, and through a 10% significance test, it indicates that the industrial structure in eastern China is excellent, which has a significant positive promoting effect on the coordinated development of the two systems in the eastern region. The central, western, and northeast regions also need to increase the development of the tertiary industry, constantly optimize the industrial structures, and enhance the positive role of the industrial structures in promoting the coordinated development of the two systems.

## 5 Suggestions and conclusions

### 5.1 Suggestions

To promote the coordinated development of traditional Chinese medicine medical treatment and social economy, it is necessary to pay attention to regional individuality based on integrity. Fully consider the regional differences of east, central, west and northeast, focusing on the improvement of quality. Form a situation of cross-regional assistance and strive to build a pattern of eastern regions helping the northeast, supporting the central region, and driving the western region.

The eastern region needs to change its resistance in terms of human capital in traditional Chinese medicine healthcare. The human capital of traditional Chinese medicine (TCM) needs to be optimized in both quantity and quality, with a reasonable allocation of TCM medical service personnel to prevent the siphoning effect of central cities on TCM medical talents and to break down the hindering role of TCM medical human capital. We need to improve the population distribution density, facilitate the two-way referral system, alleviate the excessive concentration of traditional Chinese medicine medical resources, promote resource sinking, dilute the siphoning effect, and thereby improve the utilization rate of medical and health resources, thereby enhancing the health level of the eastern region.

The human capital, transportation infrastructure and industrial structures of traditional Chinese medicine in the central region need to be improved. To maintain the strategy of the rise of central China, it is necessary to increase the support of medical and health facilities and improve the technical level of health technicians. We must improve the quality of transportation infrastructure construction in the central region and promote the transformation and upgrading of the industrial structures. The central region should actively improve the investment environment, attract high-tech talents, adjust the economic structure, and promote the transformation and upgrading of the industrial structure (48).

The western region needs to find the advantages of two systems based on resource and policy advantages. It is necessary to improve the level of economic development, strengthen the construction of transportation infrastructure, promote the introduction of medical and health talents, optimize the salary and job development structures in remote mountainous areas, and introduce more talent. By leveraging the government's policy support and fiscal orientation toward traditional Chinese medicine and economic development, we aim to enhance the level of economic development in the western region. Economically underdeveloped areas should optimize the salary and job development structure in remote mountainous areas and introduce more talent. Pay attention to the rational planning of transportation and population, and improve the accessibility of medical resources. They are actively introducing resources for industrial innovation in the eastern and central regions, enhancing their radiation and driving role in the western region, and playing a fundamental role in economic development and industrial upgrading.

The Northeast region needs to focus on improving the positive role of traditional Chinese medicine human capital, traditional Chinese medicine medical finance and population density in the coupling coordination degree of the two systems. Stimulate the internal driving force of economic development, strengthen the policy of “attracting the phoenix and returning the nest”, and ensure the return of their labor force. Raise the fertility rate of the population in northeast China and ease the degree of aging. We should increase investment in medical finance and improve the service capacity and level of traditional Chinese medicine. Continue to optimize the industrial structure, ensure the development strength of the primary and secondary industries, expand the competitive advantage of the tertiary industry, accelerate the transformation and upgrading of the secondary industry structure, extend the regional industrial chain, and stimulate the endogenous driving force of economic development.

Finally, for developing countries with unbalanced economic and medical development, governments need to formulate development policies according to local conditions. For places with stable economies, we should pay attention to maintaining their long-term growth momentum. Strengthen interregional technical cooperation in healthcare. Medical specialist alliances should be promoted among neighboring regions to promote the sinking of high-quality medical and health resources (49). More policy support must be given to less developed areas to help the strong overcome the weak and achieve common development. The reasonable planning of traffic and population in remote areas should pay attention to the accessibility of medical resources. Clarify one's own strengths and development weaknesses, and implement policies due to shortcomings. Provinces with high coupling and coordination should actively explore the path of high-level coordinated development between traditional Chinese medicine and the regional economy while maintaining their own advantages. Provinces with low coupling scheduling should clarify their own development shortcomings and characteristics, find breakthroughs in collaborative development, build a distinctive traditional Chinese medicine service system, actively seek cooperation with regions with high-coupling coordination, and focus on promoting the coupling coordination degree of each region from “competitive relationship” to “collaborative effect”.

### 5.2 Conclusion

(1) During the study period, the development level of traditional Chinese medicine medical treatment was superior to that of social and economic development, and the overall economic development was lagging behind, but the development gap between the two systems was gradually narrowing. The eastern and northeastern regions are developing synchronously, and the level of traditional Chinese medicine medical services in the central and western regions is higher than the level of economic development.

(2) The coordination degree between traditional Chinese medicine medical treatment and the social economy was mainly focused on the transitional coordination type and gradually increased steadily, but there are evident differences among different regions. The coupling coordination degree showed the development pattern of east > middle > west > northeast. The spatial agglomeration of the coupling coordination degree of the two systems in China presented a positive correlation, and the agglomeration degree was strengthened year by year.

(3) The level of economic development is the most critical factor affecting the coupling and coordination degrees of the two systems; however, there is still a need to strengthen human capital and health levels in traditional Chinese medicine. The eastern region needs to optimize the structure of traditional Chinese medicine medical human resources. The central and western regions focus on improving the level of healthy development, while the northeast region focuses on improving the level of transportation infrastructure and health. Developing countries with significant differences in coupling and coordination development should strengthen their own advantages and actively engage in mutual learning and cooperation with other high-coupling regions, strengthening cooperation and collaborative development within and outside urban agglomerations.

## 6 Limitations

On the one hand, there is currently no complete and scientific comprehensive evaluation index system for traditional Chinese medicine medical services and economic development in China, so there may be shortcomings in establishing an index system when selecting indicators in this article. The statistical data foundation related to traditional Chinese medicine medical services is weak, so this article conducts research at the provincial level in China. On the other hand, there are certain shortcomings in the selection of influencing factors in this article. Only the relatively basic influencing factors were selected, so the selection of influencing factors is not comprehensive enough. In future, based on a more complete and scientific indicator system, we will conduct in-depth research on the coordinated development of traditional Chinese medicine medical services and regional economy in various cities or urban areas of a province, making the research more in-depth and the research strategies more practical and feasible.

## Data availability statement

The original contributions presented in the study are included in the article/Supplementary material, further inquiries can be directed to the corresponding author.

## Author contributions

HL: Data curation, Software, Writing – original draft, Conceptualization. ZJ: Data curation, Investigation, Writing – review & editing. JD: Data curation, Investigation, Writing – review & editing. DL: Conceptualization, Supervision, Validation, Writing – review & editing.
