# Supplementary material for: Analysis of the coupling coordination between traditional Chinese medicine medical services and economy and its influencing factors in China
Source: Front Public Health. 2024 Jun 4;12:1320262. doi: 10.3389/fpubh.2024.1320262 (PMC11336826; doi:10.3389/fpubh.2024.1320262)
Supplement: Supplementary file 1 [file Data_Sheet_1.ZIP › supporting/Results of regional influencing factors (stata software).docx]

| Logarithm of all | The whole country | East | central | West | northeast |
| --- | --- | --- | --- | --- | --- |
| LNmp | -0.0422 | -0.111^**^ | 0.0182 | 0.0146 | -0.111^**^ |
|  | (0.031) | (0.055) | (0.045) | (0.053) | (0.055) |
| LNgow | 0.0109^***^ | 0.00700 | 0.0426^***^ | 0.00800^*^ | 0.00700 |
|  | (0.003) | (0.005) | (0.012) | (0.004) | (0.005) |
| LNpop | 0.0329^***^ | 0.0231 | 0.176^***^ | 0.0375^***^ | 0.0231 |
|  | (0.012) | (0.056) | (0.061) | (0.013) | (0.056) |
| LNpcd | 0.141^***^ | 0.163^***^ | 0.103^**^ | 0.0829 | 0.163^***^ |
|  | (0.030) | (0.055) | (0.041) | (0.055) | (0.055) |
| LNinf | 0.00812 | 0.148^**^ | 0.00722 | 0.00434 | 0.148^**^ |
|  | (0.012) | (0.063) | (0.060) | (0.011) | (0.063) |
| LNpdr | -0.107^***^ | 0.00550 | -0.0129 | -0.0981^**^ | 0.00550 |
|  | (0.029) | (0.073) | (0.059) | (0.041) | (0.073) |
| LNindu | 0.00685 | 0.0110^*^ | 0.0171 | 0.00186 | 0.0110^*^ |
|  | (0.004) | (0.006) | (0.035) | (0.026) | (0.006) |
| _cons | -0.870^***^ | -1.343^**^ | -1.329^**^ | -0.291 | -1.343^**^ |
|  | (0.309) | (0.569) | (0.579) | (0.551) | (0.569) |
| sigma_u | 0.0792^***^ | 0.114^***^ | 0.0337^***^ | 0.0552^***^ | 0.114^***^ |
|  | (0.010) | (0.028) | (0.011) | (0.013) | (0.028) |
| sigma_e | 0.0269^***^ | 0.0246^***^ | 0.0188^***^ | 0.0242^***^ | 0.0246^***^ |
|  | (0.001) | (0.002) | (0.002) | (0.002) | (0.002) |
| N | 217 | 70 | 42 | 84 | 70 |
| R^2^ | 0.940 | 0.985 | 0 .942 | 0.937 | 0.996 |

Standard errors in parentheses

^*^ *p* < 0.1, ^**^ *p* < 0.05, ^***^ *p* < 0.01

**Note:** This table is the result of regional summary. LNmp, LNpcd and LNinf represent TCM human capital, economic development level and industrial structure respectively. LNmp, LNpcd, and LNinf are the symbols used in the initial software calculation.
